# Novel or No Novel

**Published:** 1887-05-14

**Authors:** Louisa Twining


### Novel or No Novel?

BY MISS LOUISA TWINING.

As further discussion is invited on a subject on which I have already expressed opinions, I beg to be allowed to add a few remarks.

It is quite a mistake to suppose (as has been said) that I and those who agree with me in objecting to the style of tales or novels that have been introduced into THE HOSPITAL, take any ascetic or narrow view of the profession of nursing. For myself, I utterly disclaim any such view. I know and honour nurses of all sorts of opinions and classes, and consider that training and character are the two essential points to be considered with regard to their fitness.

Allow me also to say that no one enjoys a good work of fiction more than I do. That has been another supposition which I deny, that we would exclude all such books from our nurses. A library I have recently provided for infirmary nurses would at any rate prove that this is untrue. But need the scenes of all such tales, in order to be attractive to nurses, be laid in the hospitals where they work?—for this is the real point of our objection, which has been entirely overlooked by your correspondents. We are hardly so ignorant of human nature as to suppose that our nurses will not marry; no one could imagine that we were so foolish or unpractical. But, as I have said before, and will now repeat for the last time, it is the persistent connection of "hospital" and "romance" to which we object,—a connection which encourages young women to suppose that sick-wards are fit places for what, I am convinced, every hospital manager will agree with me in saying, had better be kept outside, and not looked for as what may be naturally expected there, both by doctors and nurses. With regard to some of the scenes described, I have no hesitation in saying that were they enacted in real life and by any of our own acquaintance, they would be pronounced to be highly objectionable. I can only repeat my deep regret that such possibilities should be put before the minds and thoughts of our young nurses, who, at least during the performance of their serious and responsible duties, should have far different aims and aspirations set before them.

